# Supplementary material for: STI testing and subsequent clinic attendance amongst test negative asymptomatic users of an internet STI testing service; one-year retrospective study
Source: PLoS One. 2023 Feb 8;18(2):e0281359. doi: 10.1371/journal.pone.0281359 (PMC9907837; doi:10.1371/journal.pone.0281359)
Supplement: Attachment — Submitted filename: Response to reviewer 2022 09 14.docx [file pone.0281359.s002.docx]

Response to reviewer’s comment

We thank the reviewer for their comments and have revised the manuscript to address these as highlighted in the marked manuscript. Please find below our responses to the comments

I think the paper is well written, the methods are sound and appropriate conclusion points are drawn from the data used. However, my main concern is the follow up period used. By using one calendar year of data there is unequal follow up time for testers which is a significant limitation and is not mentioned in the limitations. Additionally, at the time of the study I believe only gay, bisexual and other men who have sex with men were recommended to test 3 monthly, all sexually active men and women aged 15-24 should be tested for chlamydia annually or on change of sexual partner (whichever is more frequent). Therefore, the people who reattend within 90 days would be a select group of people who may be at higher STI risk and the results might not be generalisable to other populations. I would suggest the explain the rationale for using a 90 day cut offs or use a longer time period for follow up in order to be representative of recommended testing guidelines.

*Response: The study was designed to explore re-attendance to clinic within a short period of an initial test (‘double dipping’), as this could indicate if STI testing needs were being met by the online testing service. Given that the guideline at the time recommended high risk groups retest 3 monthly, a follow-up period more than 3 months would have limited our ability to explore the study aim, as re-attendance particularly from high risk groups could have been mostly due to routine appointments. We agree that those re-attending within 3 months may represent a high risk group but our aim was to compare re-attendance rates, for internet versus clinic testing, in asymptomatic individuals who requested a STI screen and believe that our findings remain valid. We apologise if this was not clear in the manuscript and have made additional statements in the manuscript to improve clarity, lines 114-118. We have now also highlighted the unequal follow up time period as a limitation, lines 334-337.*

Additionally, the references to the proportion of asymptomatic users of online testing services need to be clearly caveated that this metric will be biased because most people who are symptomatic are asked to go to a clinic and not use self-sampling. Especially as some services are only commissioned for asymptomatic people only.

*Response: We thank the reviewer for this observation, we have clarified this in the Discussion (lines 268-270) – ‘probably reflecting guidance advising those with symptoms to attend clinic for a full evaluation or patient preference for a clinic appointment when symptoms are present’. Also, in the method section, lines 129-131 we state, “Statistical analyses on the utilization of online STI testing were stratified by symptom presentation at the time of test request, as symptomatic patients using the Umbrella sexual health service were encouraged to attend clinic for testing”*

Specific comments
• In some parts you mention the risk of subsequent attendance. Odds ratios do not indicate the ‘risk’ but the likelihood of something occurring.

*Response: We agree with the reviewer and have rephrased the statements where ‘risk’ was used to now infer likelihood.*

• There are several places where acronyms are not spelt out

*Response: Thank you, acronyms have been spelt out.*

• Tables – there are some formatting issues; missing (95%CI), not sure why some cells are in bold.

*Response: Tables have been revised and additional table legends included for clarity.*

• Limitations - have not mentioned that you analysis will not include people attending other sexual health services.

*Response: We have commented on this limitation, line 324.*

• Point to comment on, lines 260-1: “those self-identifying as being of ‘Black ethnicity’ were significantly less likely to use the online STI test service.” Some studies found that self-sampling was difficult for some communities/ethnic groups where the test being delivered to a home address with other family members who lack of privacy. Could use this references to explain this. For example the link below <https://bmcpublichealth.biomedcentral.com/articles/10.1186/s12889-018-5256-5>

*Response: Thank you, we have made comments with respect to findings from this article, lines 273-7.*
